# Supplementary material for: Long Pentraxin 3 as a New Biomarker for Diagnosis of Hip and Knee Periprosthetic Joint Infections
Source: J Clin Med. 2023 Jan 29;12(3):1055. doi: 10.3390/jcm12031055 (PMC9917747; doi:10.3390/jcm12031055)
Supplement: Supplementary file 1 [file jcm-12-01055-s001.zip › jcm-2129820-supplementary.pdf]

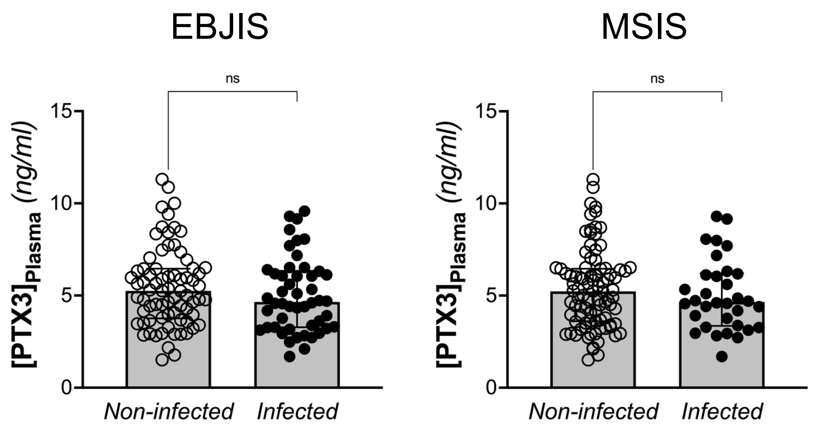


**Figure S1.** Concentration of PTX3 in the plasma of THA and TKA patients with and without PJI, based on EBJIS and MSIS criteria (Mann-Withney test, ns p>0.05).

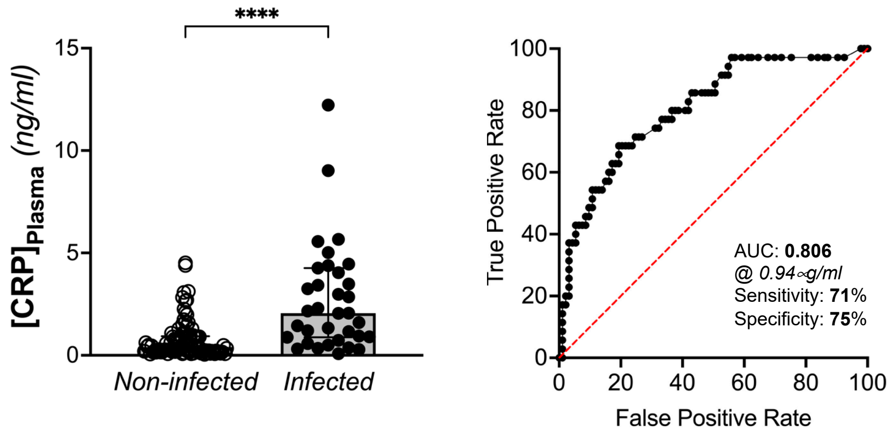


**Figure S2.** Distribution and ROC curve of the concentration of CRP in the plasma of THA and TKA patients with and without PJI, according to MSIS criteria (Mann-Withney test, **** p<0.0001).

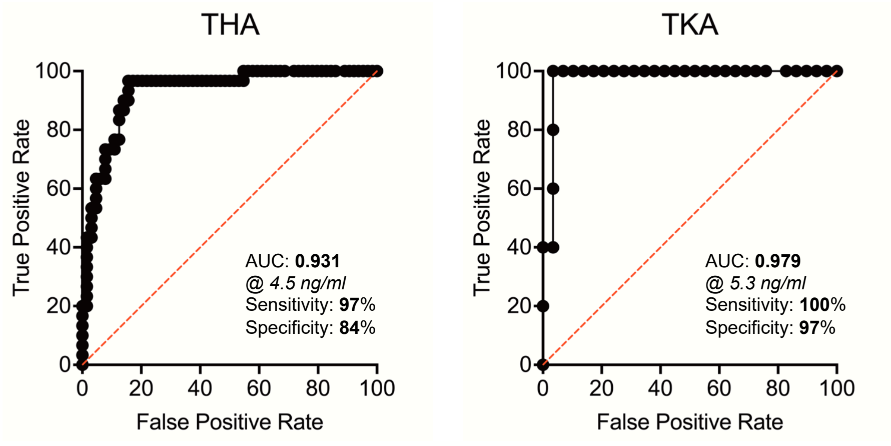


**Figure S3.** ROC curves of the concentration of PTX3 in the synovial fluid of patients with and without PJI according to surgery (THA or TKA) and MSIS criteria.

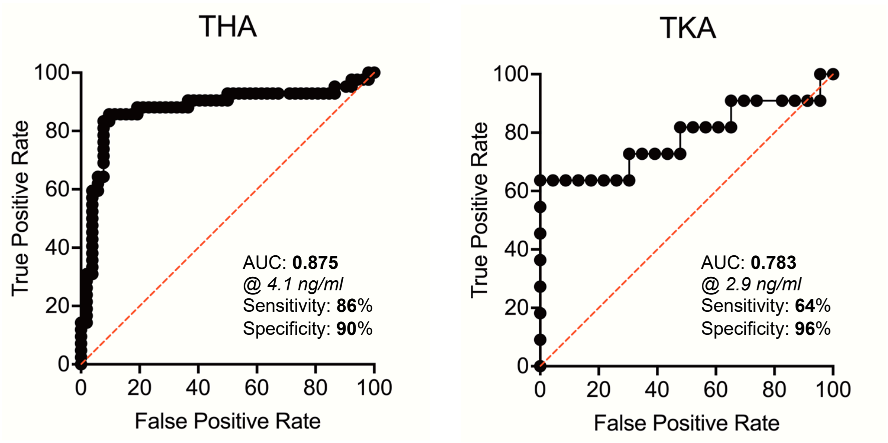


**Figure S4.** ROC curves of the concentration of PTX3 in the synovial fluid of patients with and without PJI according to surgery (THA or TKA) and EBJIS criteria.

**Table S1.** ROC curve analysis of synovial PTX3 in THA and TKA patients.

| **MSIS** | **Obs**[a] | **Threshold**[b] | **Sensitivity** | **Specificity** | **Accuracy** | **SE**[c] | **LR+**[d] | **LR-**[e] | **AUC** | **p** |
|---|---|---|---|---|---|---|---|---|---|---|
| **Synovial PTX3 in THA** *(ng/mL)* | 94 | 4.5 | 96.67% | 84.38% | 88.3% | 0.03 | 6.19 | 0.04 | 0.93 *(0.88-0.98)*[f] | <0.0001 |
| **Synovial PTX3 in TKA** *(ng/mL)* | 34 | 5.3 | 100% | 96.55% | 97.06% | 0.02 | 29 | 0 | 0.98 *(0.93-1.00)* | 0.0007 |

| **EBJIS** | **Obs** | **Threshold** | **Sensitivity** | **Specificity** | **Accuracy** | **SE** | **LR+** | **LR-** | **AUC** | **p** |
|---|---|---|---|---|---|---|---|---|---|---|
| **Synovial PTX3 in THA** *(ng/mL)* | 94 | 4.1 | 85.71% | 90.38% | 87.23% | 0.04 | 8.67 | 0.18 | 0.88 *(0.79-0.96)* | <0.0001 |
| **Synovial PTX3 in TKA** *(ng/mL)* | 34 | 2.9 | 63.64% | 95.65% | 85.30% | 0.10 | 14.64 | 0.38 | 0.78 *(0.58-0.98)* | 0.009 |

[a] Number of observations; [b] *ng/mL*; [c] Standard Error; [d] Positive likelihood ratio; [e] Negative likelihood ratio; [f] Area *(95% CI)*

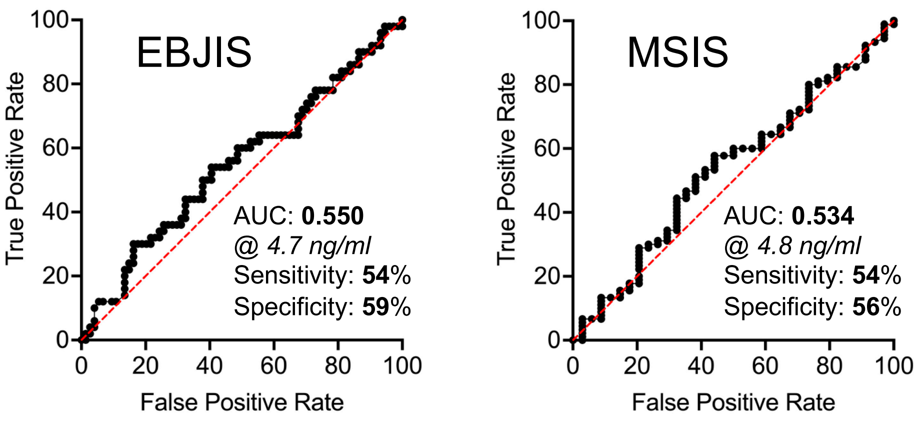


**Figure S5.** ROC curves of the concentration of PTX3 in the plasma of THA and TKA patients with and without PJI (based on EBJIS and MSIS criteria).
